# Dilead(II) hydrogen­phosphite dinitrate

**DOI:** 10.1107/S1600536809014469

**Published:** 2009-04-25

**Authors:** Rachid Ouarsal, Mohammed Lachkar, Michal Dušek, Karla Fejfarová, Brahim El Bali

**Affiliations:** a Laboratoire d’Ingénierie des Matériaux Organométalliques et Moléculaires, ‘LIMOM’, Département de Chimie, Faculté des Sciences, BP 1796, Fès-Atlas, 30000 Fès, Morocco; b Institute of Physics, Na Slovance 2, 182 21 Praha 8, Czech Republic; c Laboratory of Mineral, Solid and Analytical Chemistry, ‘LCSMA’, Department of Chemistry, Faculty of Sciences, University Mohamed I, PO Box 717, 60000 Oujda, Morocco

## Abstract

In the title compound, Pb_2_(HPO_3_)(NO_3_)_2_, the two distinct Pb^2+^ ions (both with site symmetry *m*) adopt irregular PbO_10_ coordination polyhedra. The structure is completed by two distinct nitrate groups (in which one O atom and the N atom have *m* site symmetry for both ions) and an HPO_3_^2−^ anion (in which one O atom and the P and H atoms have *m* site symmetry). The connectivity of the PbO_10_, NO_3_ and HPO_3_ units in the crystal structure results in a three-dimensional network.

## Related literature

For related structures, see: Ouarsal *et al.* (2005*a* ▶,*b* ▶); Vasić *et al.* (1981 ▶). For bond-valence sum calculations, see: Brown & Altermatt (1985 ▶).

## Experimental

### 

#### Crystal data

Pb_2_(HPO_3_)(NO_3_)_2_
*M* *_r_* = 618.4
Orthorhombic, 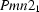

*a* = 5.4069 (2) Å
*b* = 10.4079 (6) Å
*c* = 7.1958 (4) Å
*V* = 404.94 (4) Å^3^
*Z* = 2
Mo *K*α radiation
μ = 41.76 mm^−1^
*T* = 120 K
0.25 × 0.10 × 0.05 mm

#### Data collection

Oxford Diffraction CCD diffractometer
Absorption correction: analytical (*CrysAlis RED*; Oxford Diffraction, 2008 ▶) *T*_min_ = 0.013, *T*_max_ = 0.156
6814 measured reflections
932 independent reflections
919 reflections with *I* > 3σ(*I*)
*R*_int_ = 0.026

#### Refinement

*R*[*F* ^2^ > 2σ(*F* ^2^)] = 0.012
*wR*(*F* ^2^) = 0.030
*S* = 1.25
932 reflections
78 parameters
1 restraint
Only H-atom coordinates refined
Δρ_max_ = 0.94 e Å^−3^
Δρ_min_ = −0.53 e Å^−3^
Absolute structure: Flack (1983 ▶), with 431 Friedel pairs
Flack parameter: 0.01 (1)

### 

Data collection: *CrysAlis CCD* (Oxford Diffraction, 2005 ▶); cell refinement: *CrysAlis RED* (Oxford Diffraction, 2008 ▶); data reduction: *CrysAlis RED*; program(s) used to solve structure: *SIR2002* (Burla *et al.*, 2003 ▶); program(s) used to refine structure: *JANA2006* (Petříček *et al.*, 2006 ▶); molecular graphics: *DIAMOND* (Brandenburg & Putz, 2005 ▶); software used to prepare material for publication: *JANA2006*.

## Supplementary Material

Crystal structure: contains datablocks global, I. DOI: 10.1107/S1600536809014469/hb2952sup1.cif

Structure factors: contains datablocks I. DOI: 10.1107/S1600536809014469/hb2952Isup2.hkl

Additional supplementary materials:  crystallographic information; 3D view; checkCIF report

## Figures and Tables

**Table 1:** Selected bond lengths (Å)

Pb1—O1	2.346 (6)
Pb1—O1^i^	3.042 (3)
Pb1—O3^ii^	2.418 (4)
Pb1—O4^iii^	2.884 (4)
Pb1—O6^iv^	2.881 (7)
Pb1—O6^i^	3.168 (4)
Pb2—O2^v^	2.7756 (11)
Pb2—O3^ii^	2.513 (4)
Pb2—O4^ii^	2.707 (4)
Pb2—O5^vi^	2.782 (4)
Pb2—O5^vii^	2.948 (4)

Symmetry codes: (i) 
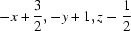
; (ii) 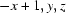; (iii) 
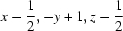
; (iv) 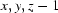; (v) 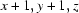; (vi) 
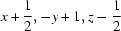
; (vii) 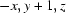.

**Fig. 1.**
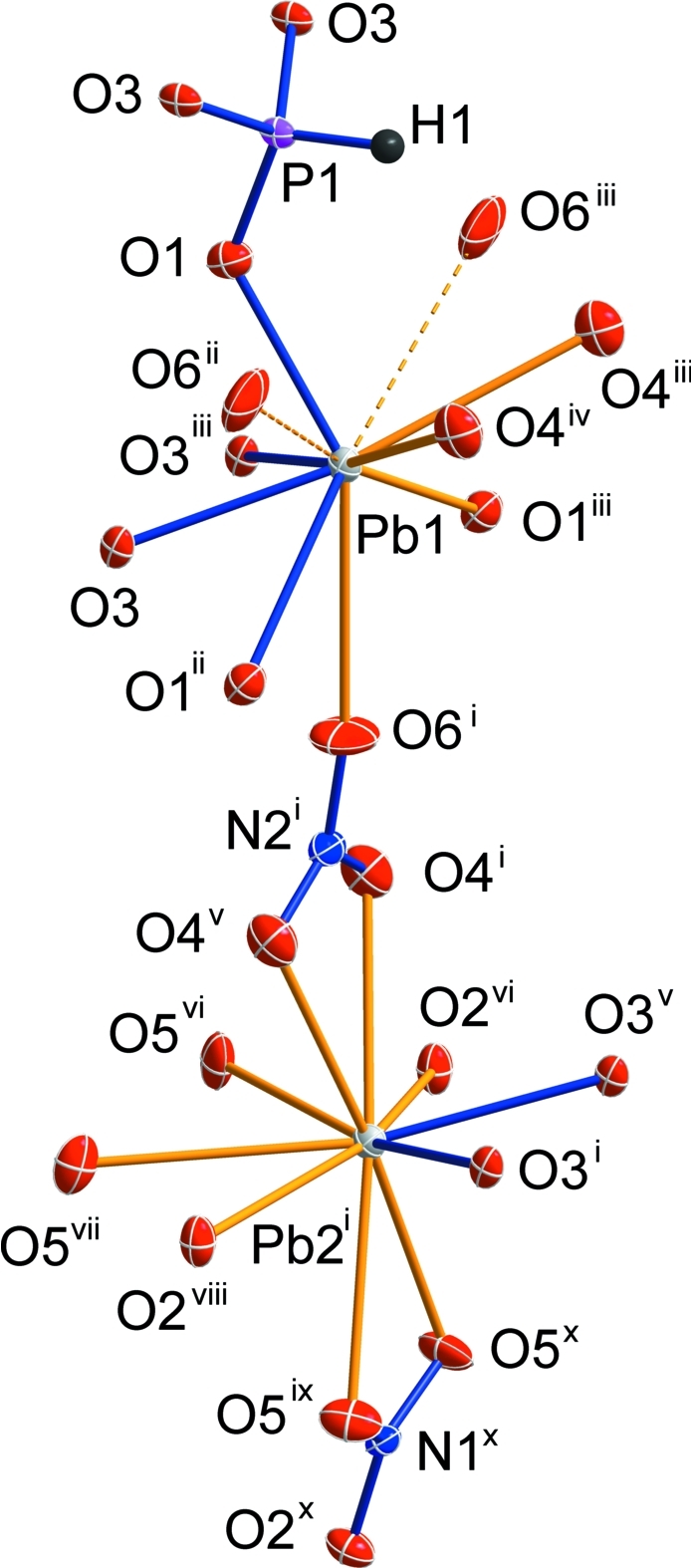
A view of the asymmetric unit and some symmetry-related atoms of (I) showing 60% probability displacement ellipsoids. The various bonds lengths are distinguished by colours and style: indigo-thick up to 2.514 Å, yellow-thick from 2.707 Å to 3.042 Å, yellow-thin-dashed for 3.168 Å. [Symmetry codes: (i) x, y, -1 + z; (ii) 1/2 - x, 1 - y, -1/2 + z; (iii) 3/2 - x, 1 - y, -1/2 + z; (iv) -1/2 - x, 1 - y, -1/2 + z; (v) 1 - x, y, -1 + z; (vi) 1 + x, 1 + y, -1 + z; (vii) -x, 1 + y, -1 + z; (viii) x, 1 + y, -1 + z; (ix) 1/2 + x, 1 - y, -3/2 + z; (x) 1/2 - x, 1 - y, -3/2 + z]

**Fig. 2.**
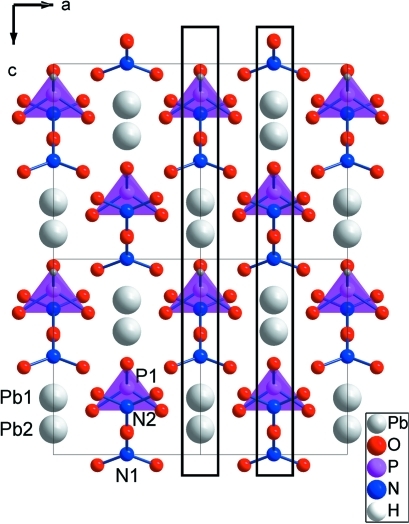
A view of the structure of the title compound along [010]. The HPO3 groups are indicated as pink tetraedra. The bonds to Pb are omitted for clarity. The black thick rectangles denote the layers that will be plotted in more details in Fig. 3.

**Fig. 3.**
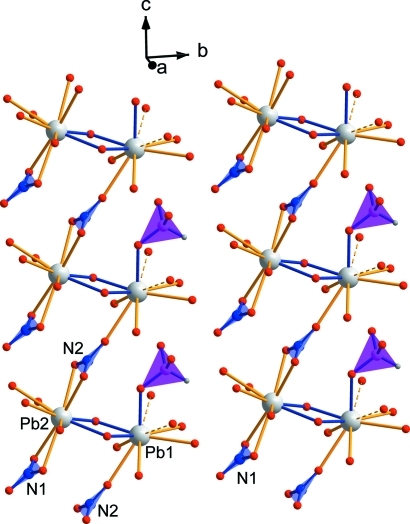
The layers indicated in Fig. 2 in a view along the a direction. The first layer (grey, blue and red atoms) is plotted together with all coordinated O atoms. The various bonds lengths are distinguished by the same way as in Fig. 1.

## supplementary crystallographic information

### Comment

As part of our ongoing structural stuides of metal hydrogenphosphites (Ouarsal *et al.* 2005*a*,*b*) we now report on the preparation and crystal structure of the title compound, (I).

The structure of (I) consists of two symmetry-independent nitrate groups and one HPO_3_ hydrogenphosphite tetrahedron. The Pb atoms are located between them in two different crystallographic positions, both of them being irregularly coordinated by ten O atoms (Fig. 1). In the coordination of Pb1, shorter bonds occur causing larger bond valence sum (Brown & Altermatt, 1985) [2.028 (10) and 1.917 (6) for Pb(1)O_10_ and Pb(2)O_10_, respectively]. On the other hand, the mean Pb—O distance is rather larger in Pb(1)O_10_ than in Pb(2)O_10_, respectively 2.835 (5) Å and 2.745 (6) Å. These values are comparable to those reported in Pb(H_2_PO_4_)_2_, 2.617 Å (Vasić *et al.*, 1981). One can distinguish the two decahedrons by their respective coordination forms: one monodentate NO_3_ and one monodentate HPO_3_ occurs in the coordination of Pb1, while two bidentate NO_3_ contribute to that of Pb2. Accordingly, the polyhedron Pb(1)O_10_ turns to be more distorted as Pb(2)O_10_.

Pb_2_(HPO_3_)(NO_3_)_2_ is characterized by a three-dimensional network. Although this is not a layer structure it is convenient from the explanatory point of view to distinguish two layers as indicated in Fig. 2. The slightly rotated view of a layer along [100] (Fig. 3) reveals strips extended in the c directions that are not interconnected within the same layer. Their connection in the b direction is mediated *via* the neighbouring layer which is shifted so that its Pb atoms fall into the holes. The shortest Pb—O bonds participate in the connection of the two PbO_10_ units and in the bond towards the HPO_3_ tetrahedron. As expected, H1 atom bonded to P is not involved in any hydrogen bonding. Average P—O distance and O—P—O and H—P—O angles are 1.531 (5) Å, 110.4 (2)° and 108.6 (2)° respectively. d_P—H_ = 1.20 (5) Å. These values lie in the same range as the ones previously reported in known phosphites (Ouarsal *et al.* 2005*b*).

### Experimental

1.6161 g lead nitrate Pb(NO_3_)_2_.6H_2_O was dissolved in 6 ml of distilled water and added to a solution of 0.4 g phosphorous acid H_3_PO_3_, dissolved in 5 ml water. The mixture was stirred for 1 h at 333 K, after which time the precipitate obtained was filtered out of the solution. The filtrate was allowed to stand at room temperature until many large, colourless needles of (I) arose. The crystals were recovered by filtration and washed with a water–ethanol (80:20 v/v) mixture.

### Refinement

The H atom was located in a difference map and its position and U_iso_ values were freely refined.

### Crystal data

**Table:**

Pb_2_(HPO_3_)(NO_3_)_2_	*F*(000) = 532
*M**_r_* = 618.4	*D*_x_ = 5.070 Mg m^−^^3^
Orthorhombic, *P**m**n*2_1_	Mo *K*α radiation, λ = 0.71069 Å
Hall symbol: P 2ac -2	Cell parameters from 6814 reflections
*a* = 5.4069 (2) Å	θ = 3.4–26.5°
*b* = 10.4079 (6) Å	µ = 41.76 mm^−^^1^
*c* = 7.1958 (4) Å	*T* = 120 K
*V* = 404.94 (4) Å^3^	Plate, colourless
*Z* = 2	0.25 × 0.10 × 0.05 mm

### Data collection

**Table:**

Oxford Diffraction CCD diffractometer	932 independent reflections
Radiation source: X-ray tube	919 reflections with *I* > 3σ(*I*)
graphite	*R*_int_ = 0.026
Detector resolution: 8.3438 pixels mm^-1^	θ_max_ = 26.5°, θ_min_ = 3.4°
Rotation method data acquisition using ω scans	*h* = −6→6
Absorption correction: analytical (*CrysAlis RED*; Oxford Diffraction, 2008)	*k* = −13→13
*T*_min_ = 0.013, *T*_max_ = 0.156	*l* = −9→9
6814 measured reflections	

### Refinement

**Table:**

Refinement on *F*^2^	Weighting scheme based on measured s.u.'s *w* = 1/[σ^2^(*I*) + 0.0004*I*^2^]
*R*[*F*^2^ > 2σ(*F*^2^)] = 0.012	(Δ/σ)_max_ = 0.013
*wR*(*F*^2^) = 0.030	Δρ_max_ = 0.94 e Å^−^^3^
*S* = 1.25	Δρ_min_ = −0.53 e Å^−^^3^
932 reflections	Extinction correction: B-C type 1 Lorentzian isotropic (Becker & Coppens, 1974)
78 parameters	Extinction coefficient: 48 (5)
1 restraint	Absolute structure: Flack (1983), with 431 Friedel pairs
1 constraint	Flack parameter: 0.01 (1)
Only H-atom coordinates refined	

### Special details

**Table:**

Refinement. The refinement was carried out against all reflections. The conventional *R*-factor is always based on *F*. The goodness of fit as well as the weighted *R*-factor are based on *F* and *F*^2^ for refinement carried out on *F* and *F*^2^, respectively. The threshold expression is used only for calculating *R*-factors *etc*. and it is not relevant to the choice of reflections for refinement.The program used for refinement, JANA2000, uses the weighting scheme based on the experimental expectations, see _refine_ls_weighting_details, that does not force *S* to be one. Therefore the values of *S* are usually larger then the ones from the *SHELX* program.

### Fractional atomic coordinates and isotropic or equivalent isotropic displacement parameters (Å^2^)

**Table:**

	*x*	*y*	*z*	*U*_iso_*/*U*_eq_	
Pb1	0.5	0.47428 (2)	0.20948	0.01003 (7)	
Pb2	0.5	0.84356 (2)	0.37011 (5)	0.01002 (8)	
O1	0.5	0.4667 (5)	0.5354 (8)	0.0147 (15)	
P1	0.5	0.34607 (15)	0.6573 (3)	0.0083 (5)	
N1	0	−0.0035 (6)	0.4972 (8)	0.0103 (17)	
O2	0	−0.0975 (5)	0.3893 (8)	0.0155 (14)	
N2	0.5	0.7144 (6)	0.7547 (8)	0.0134 (18)	
O3	0.2358 (7)	0.6561 (4)	0.2748 (5)	0.0109 (10)	
O4	0.7011 (7)	0.7548 (3)	0.6898 (6)	0.0205 (11)	
O5	−0.2007 (7)	0.0434 (3)	0.5539 (6)	0.0194 (11)	
O6	0.5	0.6317 (6)	0.8801 (10)	0.033 (2)	
H1	0.5	0.248 (4)	0.568 (10)	0.0099*	

### Atomic displacement parameters (Å^2^)

**Table:**

	*U*^11^	*U*^22^	*U*^33^	*U*^12^	*U*^13^	*U*^23^
Pb1	0.00890 (14)	0.01098 (13)	0.01022 (12)	0	0	−0.00077 (12)
Pb2	0.00891 (15)	0.01137 (12)	0.00978 (12)	0	0	−0.00032 (9)
O1	0.012 (3)	0.020 (2)	0.013 (2)	0	0	0.002 (2)
P1	0.0048 (9)	0.0110 (8)	0.0091 (9)	0	0	0.0017 (5)
N1	0.011 (3)	0.013 (2)	0.007 (3)	0	0	0.000 (2)
O2	0.012 (3)	0.015 (2)	0.019 (2)	0	0	−0.006 (2)
N2	0.019 (4)	0.011 (3)	0.010 (3)	0	0	0.000 (2)
O3	0.0055 (18)	0.0151 (16)	0.0123 (17)	−0.0002 (15)	0.0003 (14)	−0.0033 (11)
O4	0.0123 (19)	0.0226 (16)	0.027 (2)	−0.0012 (15)	−0.0004 (16)	0.0012 (17)
O5	0.013 (2)	0.0235 (17)	0.0222 (16)	0.0054 (16)	0.0001 (18)	−0.0102 (17)
O6	0.049 (4)	0.030 (3)	0.019 (3)	0	0	0.015 (3)

### Geometric parameters (Å, °)

**Table:**

Pb1—O1	2.346 (6)	Pb2—O4^iii^	2.707 (4)
Pb1—O1^i^	3.042 (3)	Pb2—O5^vii^	2.948 (4)
Pb1—O1^ii^	3.042 (3)	Pb2—O5^i^	2.782 (4)
Pb1—O3	2.418 (4)	Pb2—O5^viii^	2.782 (4)
Pb1—O3^iii^	2.418 (4)	Pb2—O5^ix^	2.948 (4)
Pb1—O4^ii^	2.884 (4)	P1—O1	1.532 (6)
Pb1—O4^iv^	2.884 (4)	P1—O3^x^	1.530 (4)
Pb1—O6^v^	2.881 (7)	P1—O3^xi^	1.530 (4)
Pb1—O6^i^	3.168 (4)	P1—H1	1.20 (5)
Pb1—O6^ii^	3.168 (4)	N1—O2	1.249 (8)
Pb2—O2^vi^	2.7756 (11)	N1—O5	1.258 (5)
Pb2—O2^vii^	2.7756 (11)	N1—O5^xii^	1.258 (5)
Pb2—O3	2.513 (4)	N2—O4	1.255 (5)
Pb2—O3^iii^	2.513 (4)	N2—O4^iii^	1.255 (5)
Pb2—O4	2.707 (4)	N2—O6	1.247 (9)
			
O1—Pb1—O1^i^	114.73 (10)	O2^vii^—Pb2—O5^i^	64.37 (14)
O1—Pb1—O1^ii^	114.73 (10)	O2^vii^—Pb2—O5^viii^	109.11 (14)
O1—Pb1—O3	80.31 (13)	O2^vii^—Pb2—O5^ix^	110.88 (13)
O1—Pb1—O3^iii^	80.31 (13)	O3—Pb2—O3^iii^	69.28 (12)
O1—Pb1—O4^ii^	91.22 (13)	O3—Pb2—O4	101.25 (12)
O1—Pb1—O4^iv^	91.22 (13)	O3—Pb2—O4^iii^	74.85 (12)
O1—Pb1—O6^v^	147.26 (18)	O3—Pb2—O5^vii^	169.07 (12)
O1—Pb1—O6^i^	66.48 (13)	O3—Pb2—O5^i^	109.08 (12)
O1—Pb1—O6^ii^	66.48 (13)	O3—Pb2—O5^viii^	83.30 (11)
O1^i^—Pb1—O1^ii^	125.41 (13)	O3—Pb2—O5^ix^	110.97 (11)
O1^i^—Pb1—O3	52.92 (13)	O3^iii^—Pb2—O4	74.85 (12)
O1^i^—Pb1—O3^iii^	116.57 (13)	O3^iii^—Pb2—O4^iii^	101.25 (12)
O1^i^—Pb1—O4^ii^	130.14 (13)	O3^iii^—Pb2—O5^vii^	110.97 (11)
O1^i^—Pb1—O4^iv^	69.42 (12)	O3^iii^—Pb2—O5^i^	83.30 (11)
O1^i^—Pb1—O6^v^	63.03 (9)	O3^iii^—Pb2—O5^viii^	109.08 (12)
O1^i^—Pb1—O6^i^	58.09 (14)	O3^iii^—Pb2—O5^ix^	169.07 (12)
O1^i^—Pb1—O6^ii^	171.25 (15)	O4—Pb2—O4^iii^	47.35 (12)
O1^ii^—Pb1—O3	116.57 (13)	O4—Pb2—O5^vii^	68.85 (11)
O1^ii^—Pb1—O3^iii^	52.92 (13)	O4—Pb2—O5^i^	133.06 (12)
O1^ii^—Pb1—O4^ii^	69.42 (12)	O4—Pb2—O5^viii^	174.94 (11)
O1^ii^—Pb1—O4^iv^	130.14 (13)	O4—Pb2—O5^ix^	94.59 (12)
O1^ii^—Pb1—O6^v^	63.03 (9)	O4^iii^—Pb2—O5^vii^	94.59 (12)
O1^ii^—Pb1—O6^i^	171.25 (15)	O4^iii^—Pb2—O5^i^	174.94 (11)
O1^ii^—Pb1—O6^ii^	58.09 (14)	O4^iii^—Pb2—O5^viii^	133.06 (12)
O3—Pb1—O3^iii^	72.44 (12)	O4^iii^—Pb2—O5^ix^	68.85 (11)
O3—Pb1—O4^ii^	171.11 (12)	O5^vii^—Pb2—O5^i^	81.63 (12)
O3—Pb1—O4^iv^	109.00 (12)	O5^vii^—Pb2—O5^viii^	106.42 (11)
O3—Pb1—O6^v^	73.43 (14)	O5^vii^—Pb2—O5^ix^	66.57 (11)
O3—Pb1—O6^i^	72.12 (14)	O5^i^—Pb2—O5^viii^	45.92 (11)
O3—Pb1—O6^ii^	134.56 (15)	O5^i^—Pb2—O5^ix^	106.42 (11)
O3^iii^—Pb1—O4^ii^	109.00 (12)	O5^viii^—Pb2—O5^ix^	81.63 (12)
O3^iii^—Pb1—O4^iv^	171.11 (12)	Pb1—O1—P1	126.8 (3)
O3^iii^—Pb1—O6^v^	73.43 (14)	O1—P1—O3^x^	109.20 (18)
O3^iii^—Pb1—O6^i^	134.56 (15)	O1—P1—O3^xi^	109.20 (18)
O3^iii^—Pb1—O6^ii^	72.12 (14)	O1—P1—H1	113 (3)
O4^ii^—Pb1—O4^iv^	68.18 (11)	O3^x^—P1—O3^xi^	112.8 (2)
O4^ii^—Pb1—O6^v^	115.45 (13)	O3^x^—P1—H1	106.4 (16)
O4^ii^—Pb1—O6^i^	102.09 (13)	O3^xi^—P1—H1	106.4 (16)
O4^ii^—Pb1—O6^ii^	41.64 (14)	O2—N1—O5	120.4 (3)
O4^iv^—Pb1—O6^v^	115.45 (13)	O2—N1—O5^xii^	120.4 (3)
O4^iv^—Pb1—O6^i^	41.64 (14)	O5—N1—O5^xii^	119.3 (5)
O4^iv^—Pb1—O6^ii^	102.09 (13)	Pb2^xiii^—O2—Pb2^xiv^	153.8 (2)
O6^v^—Pb1—O6^i^	121.12 (11)	Pb2^xiii^—O2—N1	101.78 (11)
O6^v^—Pb1—O6^ii^	121.12 (11)	O4—N2—O4^iii^	120.0 (5)
O6^i^—Pb1—O6^ii^	117.19 (15)	O4—N2—O6	120.0 (3)
O2^vi^—Pb2—O2^vii^	153.82 (14)	O4^iii^—N2—O6	120.0 (3)
O2^vi^—Pb2—O3	68.37 (13)	Pb1—O3—Pb2	108.96 (14)
O2^vi^—Pb2—O3^iii^	137.63 (13)	Pb1—O3—P1^i^	111.9 (2)
O2^vi^—Pb2—O4	115.10 (14)	Pb2—O3—P1^i^	129.5 (2)
O2^vi^—Pb2—O4^iii^	69.02 (14)	Pb1^xv^—O4—Pb2	123.29 (14)
O2^vi^—Pb2—O5^vii^	110.88 (13)	Pb1^xv^—O4—N2	101.0 (3)
O2^vi^—Pb2—O5^i^	109.11 (14)	Pb2—O4—N2	94.7 (3)
O2^vi^—Pb2—O5^viii^	64.37 (14)	Pb2^xiii^—O5—Pb2^x^	151.56 (16)
O2^vi^—Pb2—O5^ix^	44.54 (13)	Pb2^xiii^—O5—N1	93.1 (3)
O2^vii^—Pb2—O3	137.63 (13)	Pb2^x^—O5—N1	95.3 (3)
O2^vii^—Pb2—O3^iii^	68.37 (13)	Pb1^xvi^—O6—N2	171.0 (5)
O2^vii^—Pb2—O4	69.02 (14)	Pb1^xvi^—O6—O4	148.84 (17)
O2^vii^—Pb2—O4^iii^	115.10 (14)	Pb1^xvi^—O6—O4^iii^	148.84 (17)
O2^vii^—Pb2—O5^vii^	44.54 (13)		

Symmetry codes: (i) −*x*+1/2, −*y*+1, *z*−1/2; (ii) −*x*+3/2, −*y*+1, *z*−1/2; (iii) −*x*+1, *y*, *z*; (iv) *x*−1/2, −*y*+1, *z*−1/2; (v) *x*, *y*, *z*−1; (vi) *x*, *y*+1, *z*; (vii) *x*+1, *y*+1, *z*; (viii) *x*+1/2, −*y*+1, *z*−1/2; (ix) −*x*, *y*+1, *z*; (x) −*x*+1/2, −*y*+1, *z*+1/2; (xi) *x*+1/2, −*y*+1, *z*+1/2; (xii) −*x*, *y*, *z*; (xiii) *x*−1, *y*−1, *z*; (xiv) *x*, *y*−1, *z*; (xv) −*x*+3/2, −*y*+1, *z*+1/2; (xvi) *x*, *y*, *z*+1.

## References

[bb3] Brandenburg, K. & Putz, H. (2005). *DIAMOND* Crystal Impact GbR, Bonn, Germany.

[bb4] Brown, I. D. & Altermatt, D. (1985). *Acta Cryst.* B**41**, 244–247.

[bb1] Burla, M. C., Camalli, M., Carrozzini, B., Cascarano, G. L., Giacovazzo, C., Polidori, G. & Spagna, R. (2003). *J. Appl. Cryst.* **36**, 1103.

[bb6] Flack, H. D. (1983). *Acta Cryst.* A**39**, 876–881.

[bb7] Ouarsal, R., El Bali, B., Lachkar, M., Dusek, M. & Fejfarova, K. (2005*a*). *Acta Cryst.* E**61**, i168–i170. 10.1107/S1600536809014469 PMC2977541 21583727

[bb8] Ouarsal, R., El Bali, B., Lachkar, M., Dusek, M. & Fejfarova, K. (2005*b*). *Acta Cryst.* E**61**, i171–i173. 10.1107/S1600536809014469 PMC2977541 21583727

[bb9] Oxford Diffraction (2005). *CrysAlis CCD* Oxford Diffraction Ltd, Abingdon, England.

[bb12] Oxford Diffraction (2005). *CrysAlis RED* Oxford Diffraction Ltd, Abingdon, England.

[bb10] Petříček, V., Dušek, M. & Palatinus, L. (2006). *JANA2006* Institute of Physics, Prague, Czech Republic. http://www-xray.fzu.cz/jana/jana.html

[bb11] Vasić, P., Prelesnik, B., Herak, R. & Čurić, M. (1981). *Acta Cryst.* B**37**, 660–662.

